# Society and health of migrants

**DOI:** 10.1007/s10654-016-0174-2

**Published:** 2016-07-26

**Authors:** Michael Marmot

**Affiliations:** UCL Institute of Health Equity, Department of Epidemiology and Public Health, UCL, London, UK

In London, in May 2016, a photograph was tweeted with the caption: “This is what a civilised society looks like”. The photo showed the inauguration of the newly elected London mayor—hardly remarkable in itself. But the specialness of the scene grows by degrees. The mayor, Sadiq Khan, himself a civil rights lawyer, is the son of a Pakistani immigrant bus driver. It is notable that a Muslim be elected to lead a major European city. The inauguration took place in Southwark cathedral, a central place of prayer for Anglicans. Ranged behind the mayor in the photo were leaders of Catholic, Protestant, Jewish, Muslim and, I think, Hindu faiths. Also visible was Doreen Lawrence, a civil rights campaigner, whose son, a black teenager, had been murdered by white thugs.

The photo takes on even more significance given what preceded it in the election campaign. The Conservative candidate first played on divisions among South Asian communities, suggesting shamefully that Hindu wealth would be undermined by a Labour Muslim Mayor. Then, even more shamefully, suggested that the population of London might not be safe because Sadiq Khan was friendly with known supporters of terrorism and Isis. The implication was clear: the son of a Muslim immigrant was divisive and unsafe.

Khan’s multicultural, multi-faith inauguration took on even more significance given Donald Trump’s anti-immigrant bombast in the US, and his call to ban Muslim immigration. Regrettably there are also racists aplenty in Europe with far-right political parties playing on the fears and insecurities of significant proportions of the population and blaming immigrants for economic and social ills of society.

The plight of migrants, and the influence on their health of their circumstances, is of great contemporary concern. But it is not as if migration is a recent phenomenon. The Old Testament has Jacob and his family migrating to Egypt, to escape famine conditions in Canaan; and only four hundred years or so later fleeing Egypt for Canaan to escape persecution in Egypt. Being a persecuted minority has a long history. Students of the Exodus story will remember that there was a kind of enforced healthy migrant effect. The Children of Israel were in the wilderness for 40 years, until the older generation who had left slavery had all died out. It was the younger generation who crossed into Canaan.

My own studies of health of migrants, not quite of biblical antiquity, began 44 years ago. As Japanese migrated across the Pacific to Hawaii and California, their rate of stroke went down and of heart disease went up [1]. We had evidence that among men of Japanese ancestry in California the more traditional the culture the lower the heart disease risk. The challenge was to separate social and cultural influences from the more usual suspects of coronary risk factors [2].

Moving from pre-history of the 1970s to ancient history of the 1980s, we examined health by country of birth in migrants to England and Wales. There were several lessons relevant to the present [3]. First, for most countries of origin, migrants had lower mortality than people remaining behind—a healthy migrant effect. The exception was Ireland where the barriers to migration were low and, in the 1970s and 1980s, ill-health and social disadvantage might have been reasons for migration not for staying put. Second, in the early years after migration patterns of specific diseases resemble those of the old country. Over time they move closer to those of the host country. Third, the circumstances in which migrants live in the new country will affect mortality.

These early studies used the health experience of migrants to understand more about causation of disease. The papers in this issue of the journal use our knowledge of causation to understand the health of migrants [4–9]. Certainly, it is a topical issue. The UN High Commission for Refugees (UNHCR Mid year Trends 2015 http://www.unhcr.org.uk) focussing on refugees, rather than all migrants, estimates that in mid 2015 there were more than four million from Syria, 2.5 million from Afghanistan, more than one million from Somalia, and 0.5 million or more from each of South Sudan, Sudan and Democratic Republic of Congo. Most of these people, fleeing war, destitution and persecution, have ended up in Turkey or countries of the Middle East and Africa, but large numbers find their way to Europe—hence this group of papers.

Within Europe there have also been substantial movements of people from East to West. In the 2011 UK Census people were asked to state their first language. The most common first language in England, of course, was English. Number two was Polish—562,000 people. Parenthetically, there were more than 600 different responses to the question, “what is your main language”, grouped into 104 language groups. Cultural diversity is a fact of modern life and we need to be alive to the health consequences.

In the study of cardiovascular disease incidence among migrants to Denmark by Byberg et al. in this issue [4] there were an astonishing 192 nationalities represented in their cohort of migrants—the six most frequent were former Yugoslavia, Iraq, Turkey, Somalia, Thailand and Afghanistan. Importantly, the cohort distinguishes refugees (5/12ths of the whole) from family-reunified migrants (the other 7/12ths). Perhaps it comes as no surprise that CVD incidence rates are higher in the refugees than in the family-reunified migrants. For the refugees, conditions from which they escaped, the way they travelled and their circumstances in Denmark may all have been worse than for migrants reuniting with their families.

One simple indicator of degrees of disadvantage that refugees and migrants might experience is income [4]. Thirty per cent of the Danish born population have an income of more than 42,000 Euros a year; 10 % of the family-reunified migrants have this income and 5 % of the refugees. The Danish paper presents figures before and after adjustment for income and age. Before adjustment the incidence of all cardiovascular disease in female refugees is nearly double the rate for Danish born, and 50 % higher than the Danish rate in male refugees. *After* adjustment the rates in refugees were not elevated. Given that the age of the cases in refugees was similar to the Danish born population, and of refugee non-cases is likely to be lower, age should have been protecting refugees.

It means that low income is putting refugees at clear health disadvantage. We should not simply adjust away this effect of low income but recognise it as a cause of ill-health in refugees. There is then a second question which Byberg and colleagues address: after removing the effect of economic disadvantage what does health of migrants look like compared to the Danish born population? In other words, what else is going on?

The picture is varied. Family-reunified migrants had lower incidence rates of cardiovascular disease than Danish born [4]. Refugee men have higher incidence of myocardial infarction, one explanation for which is stress.

The fact that psychosocial influences might be strong in refugees is shown by the descriptive study of asylum seekers in Halle in Germany: 40 % report anxiety disorder and more than 50 % report depression [5].

The report from Ikram and colleagues on migrant mortality in six European countries emphasises the old lessons from the 1980s and before: “migrants” is too heterogeneous a group to make sweeping generalisations about health [6]. It is important to examine country of origin, country of destination and the circumstances of migration. The Ikram report allows for speculation rather than pointing to specific explanations as data to test specific causal hypotheses were limited.

One clear example of influence of country of origin is in the paper by Melhem and colleagues from Lebanon that points to the high rate of Hepatitis A infection in refugees from Syria and elsewhere [7]. Here the concern is not only to protect the refugees but to protect the host population by instituting Hepatitis A immunisation.

This proposal relates to a more general concern with which I began this commentary: the way refugees and other migrants are treated socially and economically, as well as medically, by host countries in Europe. There are some politicians who would argue that to treat migrants well is simply to encourage others to come. Such a view argues, in effect, that individuals be treated as instruments of political policy. This view is immoral. It runs counter to medical ethics that state clearly that all individuals should be treated with dignity.

One way to treat people with dignity is to understand and respond to health problems caused by their migrant status. These papers are a step in that direction.
